# Supplementary material for: Resolving the full spectrum of human genome variation using Linked-Reads
Source: Genome Res. 2019 Apr;29(4):635–45. doi: 10.1101/gr.234443.118 (PMC6442396; doi:10.1101/gr.234443.118)
Supplement: Supplemental Material [file supp_gr.234443.118_Supplemental_longranger-2.2.zip › longranger-2.2.tar.bz2!/lr2.2-public/lib/stan/cmdstan/stan/doc/api/html/agrad_2fwd_2functions_2_phi_8hpp_source.html]

Stan: src/stan/agrad/fwd/functions/Phi.hpp Source File


|  |  |
| --- | --- |
|  | Stan  2.5.0  probability, sampling & optimization |


- Main Page
- Related Pages
- Namespaces
- Classes
- Files

- File List
- File Members

All Classes Namespaces Files Functions Variables Typedefs Enumerations Enumerator Friends Macros Pages

- src
- stan
- agrad
- fwd
- functions

Phi.hpp

Go to the documentation of this file.

1 #ifndef STAN\_\_AGRAD\_\_FWD\_\_FUNCTIONS\_\_PHI\_HPP

2 #define STAN\_\_AGRAD\_\_FWD\_\_FUNCTIONS\_\_PHI\_HPP

3

4 #include <stan/agrad/fwd/fvar.hpp>

5 #include <stan/meta/traits.hpp>

6 #include <stan/math/functions/Phi.hpp>

7 #include <stan/math/constants.hpp>

8

9 namespace stan {

10

11  namespace agrad {

12

13  template <typename T>

14  inline fvar<T> Phi(const fvar<T>& x) {

15  using stan::math::Phi;

16  using std::exp;

17  using std::sqrt;

18  T xv = x.val\_;

19  return fvar<T>(Phi(xv),

20  x.d\_ \* exp(xv \* xv / -2.0) / sqrt(2.0 \* stan::math::pi()));

21  }

22  }

23 }

24 #endif

stan::agrad::Phi

fvar< T > Phi(const fvar< T > &x)

**Definition:** Phi.hpp:14

stan::agrad::fvar

**Definition:** fvar.hpp:13

Phi.hpp

traits.hpp

stan::math::Phi

boost::math::tools::promote\_args< T >::type Phi(const T x)

The unit normal cumulative distribution function.

**Definition:** Phi.hpp:31

stan::agrad::sqrt

fvar< T > sqrt(const fvar< T > &x)

**Definition:** sqrt.hpp:15

fvar.hpp

stan::agrad::fvar::d\_

T d\_

**Definition:** fvar.hpp:16

stan::math::pi

double pi()

Return the value of pi.

**Definition:** constants.hpp:77

constants.hpp

stan::agrad::exp

fvar< T > exp(const fvar< T > &x)

**Definition:** exp.hpp:16

stan::agrad::fvar::val\_

T val\_

**Definition:** fvar.hpp:15


---

[ Stan Home Page ]

*© 2011–2014,
Stan Development Team.*
